# Supplementary material for: Pyruvate Dehydrogenase Kinase 1 inhibition mediated oxidative phosphorylation enhancement in cartilage promotes osteoarthritis progression
Source: BMC Musculoskelet Disord. 2023 Jul 20;24:597. doi: 10.1186/s12891-023-06585-6 (PMC10357736; doi:10.1186/s12891-023-06585-6)
Supplement: Supplementary file 1 — Additional file 1. [file 12891_2023_6585_MOESM1_ESM.zip › KEGG.Gsea.1653622432464/KEGG_PRIMARY_IMMUNODEFICIENCY.html]

Details for gene set KEGG\_PRIMARY\_IMMUNODEFICIENCY[GSEA]

|  || Dataset | 55457\_collapsed\_to\_symbols.cls.cls #OA\_versus\_normal.cls.cls #OA\_versus\_normal\_repos |
| Phenotype | cls.cls#OA\_versus\_normal\_repos |
| Upregulated in class | OA |
| GeneSet | KEGG\_PRIMARY\_IMMUNODEFICIENCY |
| Enrichment Score (ES) | 0.18473808 |
| Normalized Enrichment Score (NES) | 0.6929139 |
| Nominal p-value | 0.9388235 |
| FDR q-value | 1.0 |
| FWER p-Value | 1.0 |
Table: GSEA Results Summary

  

Fig 1: Enrichment plot: KEGG\_PRIMARY\_IMMUNODEFICIENCY      
 Profile of the Running ES Score & Positions of GeneSet Members on the Rank Ordered List

  

| SYMBOL | TITLE | RANK IN GENE LIST | RANK METRIC SCORE | RUNNING ES | CORE ENRICHMENT || 1 | BLNK | B cell linker [Source:HGNC Symbol;Acc:HGNC:14211] | 546 | 0.643 | 0.0248 | Yes |
| 2 | BTK | Bruton tyrosine kinase [Source:HGNC Symbol;Acc:HGNC:1133] | 889 | 0.545 | 0.0553 | Yes |
| 3 | RFX5 | regulatory factor X5 [Source:HGNC Symbol;Acc:HGNC:9986] | 1343 | 0.451 | 0.0672 | Yes |
| 4 | RFXANK | regulatory factor X associated ankyrin containing protein [Source:HGNC Symbol;Acc:HGNC:9987] | 1383 | 0.445 | 0.1107 | Yes |
| 5 | AICDA | activation induced cytidine deaminase [Source:HGNC Symbol;Acc:HGNC:13203] | 1825 | 0.381 | 0.1163 | Yes |
| 6 | LCK | "LCK proto-oncogene, Src family tyrosine kinase [Source:HGNC Symbol;Acc:HGNC:6524]" | 2274 | 0.329 | 0.1160 | Yes |
| 7 | CD79A | CD79a molecule [Source:HGNC Symbol;Acc:HGNC:1698] | 2288 | 0.328 | 0.1493 | Yes |
| 8 | CD19 | CD19 molecule [Source:HGNC Symbol;Acc:HGNC:1633] | 2757 | 0.274 | 0.1416 | Yes |
| 9 | CD3D | CD3d molecule [Source:HGNC Symbol;Acc:HGNC:1673] | 2771 | 0.273 | 0.1691 | Yes |
| 10 | CD3E | CD3e molecule [Source:HGNC Symbol;Acc:HGNC:1674] | 2919 | 0.259 | 0.1847 | Yes |
| 11 | RFXAP | regulatory factor X associated protein [Source:HGNC Symbol;Acc:HGNC:9988] | 3624 | 0.191 | 0.1500 | No |
| 12 | CD8B | CD8b molecule [Source:HGNC Symbol;Acc:HGNC:1707] | 4226 | 0.135 | 0.1174 | No |
| 13 | CD4 | CD4 molecule [Source:HGNC Symbol;Acc:HGNC:1678] | 4465 | 0.116 | 0.1110 | No |
| 14 | IL2RG | interleukin 2 receptor subunit gamma [Source:HGNC Symbol;Acc:HGNC:6010] | 4887 | 0.081 | 0.0867 | No |
| 15 | CD40LG | CD40 ligand [Source:HGNC Symbol;Acc:HGNC:11935] | 5529 | 0.030 | 0.0401 | No |
| 16 | UNG | uracil DNA glycosylase [Source:HGNC Symbol;Acc:HGNC:12572] | 6078 | -0.011 | -0.0014 | No |
| 17 | CD8A | CD8a molecule [Source:HGNC Symbol;Acc:HGNC:1706] | 6579 | -0.048 | -0.0353 | No |
| 18 | TAP1 | "transporter 1, ATP binding cassette subfamily B member [Source:HGNC Symbol;Acc:HGNC:43]" | 6633 | -0.052 | -0.0339 | No |
| 19 | RAG1 | recombination activating 1 [Source:HGNC Symbol;Acc:HGNC:9831] | 6806 | -0.063 | -0.0407 | No |
| 20 | CD40 | CD40 molecule [Source:HGNC Symbol;Acc:HGNC:11919] | 6825 | -0.065 | -0.0353 | No |
| 21 | ZAP70 | zeta chain of T cell receptor associated protein kinase 70 [Source:HGNC Symbol;Acc:HGNC:12858] | 6962 | -0.077 | -0.0378 | No |
| 22 | RAG2 | recombination activating 2 [Source:HGNC Symbol;Acc:HGNC:9832] | 7462 | -0.115 | -0.0646 | No |
| 23 | IGLL1 | immunoglobulin lambda like polypeptide 1 [Source:HGNC Symbol;Acc:HGNC:5870] | 7544 | -0.122 | -0.0581 | No |
| 24 | PTPRC | protein tyrosine phosphatase receptor type C [Source:HGNC Symbol;Acc:HGNC:9666] | 7744 | -0.137 | -0.0592 | No |
| 25 | ADA | adenosine deaminase [Source:HGNC Symbol;Acc:HGNC:186] | 8105 | -0.163 | -0.0701 | No |
| 26 | TNFRSF13B | TNF receptor superfamily member 13B [Source:HGNC Symbol;Acc:HGNC:18153] | 8664 | -0.206 | -0.0920 | No |
| 27 | CIITA | class II major histocompatibility complex transactivator [Source:HGNC Symbol;Acc:HGNC:7067] | 9184 | -0.246 | -0.1065 | No |
| 28 | IL7R | interleukin 7 receptor [Source:HGNC Symbol;Acc:HGNC:6024] | 9233 | -0.251 | -0.0841 | No |
| 29 | ICOS | inducible T cell costimulator [Source:HGNC Symbol;Acc:HGNC:5351] | 10320 | -0.353 | -0.1315 | No |
| 30 | JAK3 | Janus kinase 3 [Source:HGNC Symbol;Acc:HGNC:6193] | 10908 | -0.418 | -0.1335 | No |
| 31 | IKBKG | inhibitor of nuclear factor kappa B kinase regulatory subunit gamma [Source:HGNC Symbol;Acc:HGNC:5961] | 10986 | -0.428 | -0.0947 | No |
| 32 | TAP2 | "transporter 2, ATP binding cassette subfamily B member [Source:HGNC Symbol;Acc:HGNC:44]" | 11616 | -0.519 | -0.0893 | No |
| 33 | AIRE | autoimmune regulator [Source:HGNC Symbol;Acc:HGNC:360] | 12610 | -0.804 | -0.0823 | No |
| 34 | DCLRE1C | DNA cross-link repair 1C [Source:HGNC Symbol;Acc:HGNC:17642] | 12818 | -1.002 | 0.0064 | No |
Table: GSEA details [plain text format]

  

Fig 2: KEGG\_PRIMARY\_IMMUNODEFICIENCY      
 Blue-Pink O' Gram in the Space of the Analyzed GeneSet

  

Fig 3: KEGG\_PRIMARY\_IMMUNODEFICIENCY: Random ES distribution      
 Gene set null distribution of ES for **KEGG\_PRIMARY\_IMMUNODEFICIENCY**
